# Lecture upon the Organon

**Published:** 1888-01

**Authors:** T. F. Allen

**Affiliations:** New York


### HOMŒOPATHIC PHYSICIAN,

A MONTHLY JOURNAL OF MEDICAL SCIENCE.

"If our school ever gives up the strict inductive method of Hahnemann, we are lost, and deserve only to be mentioned as a caricature in the history of medicine."—CONSTANTINE HERING.

Vol. VIII. JANUARY, 1888. No. 1.

### LECTURE UPON THE *ORGANON*.

PROFESSOR T. F. ALLEN, M. D., NEW YORK.

(A stenographic report of a part of one of Professor T. F. Allen's lectures on the *Organon*.)

In our last lecture on the *Organon* we took up the consideration of Hahnemann's idea of the vital force of individuals and its relation to disease. To-day I ask your consideration of Hahnemann's theory of the spirit-like essence of drugs, which he considered to be the only means whereby the vital force of the individual could be modified, that is, in health made sick or in disease restored to health. Do not confound the words "spirit-like essence" with the idea of a spirit. It is not necessary for us to go back to the childhood of the human race and suppose that in every stick and stone and clod of earth is a spirit which determines its form and nature; but it is necessary for us to conceive of some sort of force inherent in every substance, whether animal, vegetable, or mineral, which determines its materal form and properties. Has any one of you thought for a moment of the difference between a particle of white arsenic and a particle of flour, so similar, yet so unlike? What is there in this particle of white substance which determines its poisonous property and gives it all the peculiarities which we term arsenic? It is composed of atoms and molecules similar to the atoms and molecules in any other substance, but so grouped as to give it its peculiar poisonous properties. We may subdivide these atoms and molecules to the utmost limit and still the properties of arsenic remain. If we were to destroy these molecules of arsenic would there anywhere exist a spirit-like force which animates them in a manner similar to the spirit-like force which animates our human bodies and which are able to exist independently of the molecules and atoms of which we are composed? What can determine the nature of arsenic? It may be stated that possibly the peculiar grouping of the atoms or the peculiar force which determines oscillation or orbital motions in these atoms may in some manner determine its properties and its effects upon the human body. What this force is and how it is associated with the molecules and atoms of arsenic no man can tell. Substances similar to arsenic and to other poisons may be manufactured by the chemist in his laboratory from the simple elements, may come to possess poisonous properties, that is to say, they may become endowed with a spirit-like essence. This spirit-like essence is then associated in some way with the molecules and atoms of the substance. The size of atoms and molecules is pretty well determined. It is within bounds to say that if a pin's head were magnified to a globe of the size of the earth each one of the atoms would be perfectly visible and appear to be approximately of the size of buckshot. If this be true, as stated by most natural philosophers of our time, then a centesimal dilution of a drug will somewhere about the twelfth dilution represent the extreme divisibility of an atom; in other words, one drop of the twelfth centesimal dilution will contain one atom of the original substance. After this point is reached it is probable that there is no longer any atom or molecule of the original substance.

Does, then, medicinal power of the drug cease with the twelfth dilution or thereabouts?

Hahnemann's theory of dynamization is that when a substance be subdivided and at the same time agitated either by trituration of the dry substance or by shaking of the liquid the spirit-like force of the substance is increased and thereby made more ready to act upon the vital force of the individual. It is by some also supposed that with the subdivision of the material substance and its continual agitation, the spirit-like force may be in a measure or even completely separated from the original atom or molecule and transferred to the substance with which it is triturated or shaken.

This doctrine of the transference of the spirit-like essence of drugs is to my mind unthinkable. While formerly I indorsed it as a possible working hypothesis on account of the supposed action of dilutions above the material subdivisions of atoms and molecules, yet it is so at variance with all known laws of philosophy and so incapable of actual demonstration that I have been obliged to abandon it. Shall we then entirely abandon our faith in the action of potencies above the so-called material limit?

No man living dares say that he knows of what matter consists, or where is the limit of the divisibility of matter. Atoms and molecules represent the combining properties of substances and furnish chemists a good working basis. It seems that by means of atoms and molecules matter becomes, if I may use the word, material, and these material manifestations of substances seem to be definite in different substances. But no man at the present time supposes for a moment that the atoms and molecules constitute the material basis of the universe. The truth is that atoms and molecules oscillate in large orbits in a sea of universal ether very much as fishes swim in the ocean, only their oscillations are circumscribed and definite. To explain the simple phenomenon of light it is absolutely necessary to suppose the existence of an ether which fills the whole universe, the construction of which is as much more subtle than that of atoms as that of the fish is more gross than the atoms of the water in which he swims. It is necessary to suppose that this ether is an elastic solid, that an impression at any one point of the universe pervades the whole ether to its extremest point; more than this, it is impossible at the present time to explain the laws of electricity except on the supposition that it is a fluid permeating the whole universe, permeating this elastic ether very much as water permeates jelly.

So, gentlemen, when a man or a body of men resolves that the subdivision of matter ceases with the tenth centesimal dilution or with any other dilution with which we are acquainted, it seems to me, and it seems to you, to be the ignorant expression of prejudiced minds. It is absolutely impossible at the present day to know of what matter consists, or what are its limits of divisibility, just as impossible as it to know what force is or how it is united with matter, or whether it be not, indeed, identical with matter.

I speak of these things simply to caution you against bigotry and narrow-mindedness, to express the hope that your minds will ever be open to conviction, ever ready to receive the truth from whatever source it may come.

In former years I have expressed my belief in the actual power of drugs in potencies as high as the thirtieth, two hundredth, or even higher, to cure disease, but on attempting the demonstration of the effects of these potencies upon healthy individuals, I have confessed in public that I have been unable to do it, and I have also stated my conviction that the treatment of the sick with potencies well within the material range is quite as successful, or even more so, than the treatment of the sick with potencies above this range. But I would be the last to declare that because I have failed to demonstrate the power of the thirtieth and two hundredth on healthy people to my satisfaction, therefore there can be no good in them; this would be an wholly indefensible and unwarrantable position to take. I can only tell you to-day when asked the question, that I do not know, nor does anybody know; the question has not been proved; it is still an open one.

I will now pass to the consideration of another topic from Hahnemann's *Organon*, and that is the controllable power of drugs over disease. It is distinctly stated in paragraph 26, that in order to cure disease, the natural disease must be replaced by an artificial one which shall be somewhat stronger, strong enough to convert it into an artificial disease. In order to do this we must prescribe a drug which produces symptoms exactly similar to those of the patient, for two exactly similar diseases cannot coexist in the same individual. We have, then, to administer to our patient a dose of the drug strong enough to extinguish the disease, but not so strong as to create an unnaturally violent new disease, that is, to produce a violent aggravation. Drug action is speedily repelled by the vital force of the individual, and if a drug aggravation be produced, it is speedily overcome, should it not be too violent, and a rapid recovery results. * * * * * *

